# Headspace Solid-Phase Microextraction/Gas Chromatography–Mass Spectrometry and Chemometric Approach for the Study of Volatile Profile in X-ray Irradiated Surface-Ripened Cheeses

**DOI:** 10.3390/foods13030416

**Published:** 2024-01-27

**Authors:** Carmen Palermo, Annalisa Mentana, Michele Tomaiuolo, Maria Campaniello, Marco Iammarino, Diego Centonze, Rosalia Zianni

**Affiliations:** 1 Dipartimento di Medicina Clinica e Sperimentale, Università di Foggia, Via Napoli 25, 71122 Foggia, Italy; carmen.palermo@unifg.it; 2 Laboratorio Nazionale di Riferimento per il Trattamento degli Alimenti e dei Loro Ingredienti con Radiazioni Ionizzanti, Istituto Zooprofilattico Sperimentale della Puglia e della Basilicata, Via Manfredonia 20, 71121 Foggia, Italy; michele.tomaiuolo@izspb.it (M.T.); maria.campaniello@izspb.it (M.C.); marco.iammarino@izspb.it (M.I.); rosalia.zianni@izspb.it (R.Z.); 3 Dipartimento di Scienze Mediche e Chirurgiche, Università di Foggia, Via Napoli 25, 71122 Foggia, Italy; diego.centonze@unifg.it

**Keywords:** food irradiation, food safety, surface-ripened cheeses, volatile organic compounds (VOCs), HS-SPME/GC–MS, experimental design, chemometric analysis

## Abstract

X-ray irradiation is an emerging non-thermal technology that is used as a preservation and sanitization technique to inactivate pathogens and spoilage organisms, increasing the shelf life of products. In this work, two different types of surface-ripened cheeses, Brie and Camembert, produced with cow milk, were treated with X-rays at three dose levels, 2.0, 4.0 and 6.0 kGy, to evaluate the irradiation effects on the volatile profile using a volatolomic approach. The headspace solid-phase microextraction (HS-SPME) technique combined with gas chromatography–mass spectrometry (GC–MS) was used to extract and analyze the volatile fraction from these dairy matrices. The HS-SPME method was optimized by a central composite design in combination with a desirability optimization methodology. The Carboxen/PDMS fiber, 50 °C for extraction temperature and 60 min for time extraction were found to be the best parameter settings and were applied for this investigation. The obtained fingerprints demonstrated that the irradiation-induced changes are dose dependent. The X-ray irradiation produced many new volatiles not found in the non-irradiated samples, but it also varied the amount of some volatiles already present in the control. Specifically, aldehydes and hydrocarbons increased with the irradiation dose, whereas alcohols, carboxylic acids, esters, methyl esters, ketones, lactones and sulfur-containing compounds showed a non-linear dependence on the dose levels; indeed, they increased up to 4.0 kGy, and then decreased slightly at 6.0 kGy. This trend, more evident in the Camembert profile, is probably due to the fact that these compounds are involved in different oxidation mechanisms of lipids and proteins, which were induced by the radiation treatment. In these oxidative chemical changes, the production and degradation processes of the volatiles are competitive, but at higher doses, the decomposition reactions exceed those of formation. A principal component analysis and partial least square discriminant analysis were used to discriminate between the treated and untreated samples. Moreover, this study allowed for the identification of potential markers of X-ray treatment for the two cheeses, confirming this approach as a useful tool for the control of irradiated surface-ripened cheeses.

## 1. Introduction

Cheeses ripened by molds are divided into two broad categories: those ripened with surface mold (e.g., Camembert and Brie) and blue vein cheeses (e.g., Roquefort and Gorgonzola) [1]. Brie and Camembert cheeses, both originating from France and very popular in Europe, are largely achieved with *Penicillium caseicolum*, although, historically, Camembert was manufactured with the closely-related *Penicillium camemberti* [2]. These two surface-ripened cheeses share commonalities yet exhibit distinct characteristics. Brie tends to be larger in size with a subtle, buttery flavor and a slightly firmer texture. Its rind is thin and edible, showcasing a mellow aroma. On the other hand, Camembert is smaller with a richer taste that verges on earthy and mushroom-like. Its rind is thicker and possesses a more pungent aroma. These variations arise due to factors such as aging time, production methods, the regions they originate from and above all, the biodiversity of the fungi growing within [3,4]. The white surface mold provides a unique appearance, aroma and taste, but on the other hand, it is a perfect media for the multiplication of bacteria such as *Listeria monocytogenes* [5,6]. Moreover, Brie and Camembert cheeses are included in the “ready-to-eat” food category, and together with other soft cheeses, have been incriminated for listeriosis outbreaks around the world [7]. Traditionally, the microbiological safety of cheeses is assured by thermal pasteurization, which, unfortunately, is insufficient to destroy or prevent the growth of all pathogenic, spoilage or spore-forming bacteria. Even if post-pasteurization contamination can occur, it can lead to increased bacterial levels and spoilage that can be detected sensorially [8]. Food irradiation consists of direct exposure to γ-rays, e-beams or X-rays in a controlled environment [9]. The gray (Gy) is the unit of ionizing radiation dose in the International System of Units and is defined as the absorption of 1 joule of radiation energy per kilogram of matter. Doses of thousands of Gy (kilogray, kGy) are used for food irradiation and the recommended dose of X-rays should not exceed 10 kilogray (kGy) [10]. Treatment with ionizing radiations as a method for decontaminating, destroying and reducing the overall microbial load is a valid alternative to traditional thermal procedures, which are often not sufficient or ineffective [11]. In 1992, the European Committee established that the treatment of Camembert cheese, produced with raw milk, with ionizing radiation at doses of up to 2.5 kGy is acceptable from a health point of view, thus inserting it in the list of food classes authorized for irradiation [12]. To date, in accordance with the European regulatory framework, only this cheese can be irradiated. In France, Camembert cheese can be irradiated with doses from 2.25 kGy to 3.5 kGy [5]. Specifically, in accordance with European legislation, the dose levels recommended for dairy products are values less than or equal to 3.0 kGy to prevent rancidity and off-flavors [9,13]. The principal aim of the research in the field of food irradiation is the evaluation of chemical changes and the consequent determination of treatment markers. Indeed, ionizing radiation leads to radiolysis and/or the oxidation of lipids and proteins through the direct interactions of the radiation with these macromolecules or indirect interactions via the products of H_2_O radiolysis [14,15]. Among these radiation-induced chemical changes, there is the production of volatile organic compounds (VOCs), which is dependent on both the dose level and on the presence of O_2_ [14,15,16]. Hence, specific VOCs, such as hydrocarbons, aldehydes, ketones, esters, alcohols and sulfur-containing compounds, could be potential markers for several foodstuffs such as meats [16,17,18], fish [19,20,21] and fruits [22,23]. To date, detailed studies in dairy products on VOC behavior as a result of the irradiation treatment and dose level are still lacking [24,25]. The application of irradiation to cheeses, with related insights, has received little attention, mainly due to the complexity of the product varieties [9,24].

The aim of this work was to identify the modifications to the volatile profile as a consequence of X-ray irradiation in surface mold-ripened cheeses, such as Brie and Camembert, using three different dose levels of 2.0, 4.0 and 6.0 kGy. Specifically, the 2.0 kGy value is within the range of authorized doses. The higher doses of 4.0 and 6.0 kGy were considered to verify what kind of chemical modifications can occur and how they can affect the organoleptic properties of treated cheeses at non-conventional levels. Headspace solid-phase microextraction (HS-SPME) coupled with gas chromatography–mass spectrometry (GC–MS) was used to analyze the volatile profiles of Brie and Camembert cheeses to study the effects of X-ray irradiation treatment. The Central Composite Design (CCD) in combination with a Desirability Optimization Methodology (DOM) was applied for the optimization of the HS-SPME procedure in terms of the type of fiber, extraction temperature and extraction time. A Principal Component Analysis (PCA) and classification models of Partial Least Square Discriminant Analysis (PLS-DA) were used to discriminate between the treated and untreated samples. To the best of our knowledge, this is the first time that the HS-SPME procedure has been optimized to analyze the aroma profiles of X-ray-irradiated Brie and Camembert cheeses using an experimental design. Furthermore, volatolomic and chemometric approaches were used to characterize the quality-type fingerprints of the two cheeses, considering the irradiation treatment and the dose levels.

## 2. Materials and Methods

### 2.1. Chemical Standards and Sample Preparation

Methanol, 3–octanol, used as internal standard (IS), and C7–C30 saturated n-alkane mixture (1000 mg/L) were purchased from Merck Life Science S.r.l. (Darmstadt, Germany). Brie and Camembert cheese samples of 250 g, produced from pasteurized cow milk, were acquired in a local market, and stored at 4 °C. For both cheeses, one sample was used as a control and the others were irradiated separately. Aliquot of thin slices of 0.5 g, also containing the white rinds, were weighed in a 20 mL screw glass headspace vials, spiked with 25 µL of IS (IS, 20 mg/L in methanol) and then sealed with a cap equipped with a silicone/PTFE septum (Supelco, Bellefonte, PA, USA) for HS-SPME/GC–MS analysis.

### 2.2. Irradiation Treatment

X-ray irradiation treatment was performed at the National Reference Laboratory for treatment of irradiated foods and their ingredients (Istituto Zooprofilattico Sperimentale della Puglia e della Basilicata, Foggia, Italy) with a biological X-ray irradiator (RS–2400, Radsource Inc., Suwanee, GA, USA). Two Brie samples were irradiated at 4.0 kGy, homogenized, and subsequently aliquoted for CCD development. For Brie and Camembert cheeses, one sample per dose level (2.0, 4.0 and 6.0 kGy) was irradiated at room temperature (25  ±  2 °C). Samples were rotated 360° continuously during the irradiation process to achieve uniform target doses. The doses given to the samples were measured by an alanine/electron paramagnetic resonance dosimetry system [26]. Aliquots (0.5 g) of irradiated Brie and Camembert were analyzed by HS-SPME/GC–MS immediately after X-ray irradiation.

### 2.3. Headspace Solid-Phase Microextraction (HS-SPME) and Gas Chromatography–Mass Spectrometry (GC–MS)

Two fibers were tested for CCD: 50/30 μm divinylbenzene/Carboxen/polydimethylsiloxane (DVB/CAR/PDMS) and 85 μm Carboxen/polydimethylsiloxane (Carboxen/PDMS) (Supelco, Bellefonte, PA, USA). These fibers were thermally conditioned before first use.

A Gerstel MPS auto-sampler (Baltimore, MD, USA) and an Agilent 6890 N gas chromatograph coupled with an Agilent 5975 mass selective detector (Little Falls, DE, USA) were used for HS-SPME/GC–MS analyses. Data were acquired using MSD ChemStation software (Agilent, Little Falls, DE, USA). All GC–MS operating conditions were described in our previous work [24]. The experimental linear retention indices (LRI_E_) of VOCs were calculated from n-alkane standards (C7–C30) by the Van Den Dool formula [27]. VOC identification was carried out by comparing their mass spectra with those in the NIST 05 mass spectral library (National Institute of Standards and Technology, Gaithersburg, MD, USA) with matching probability >85% and by comparing their LRI_E_ with the reference indices (LRI_R_), available in PubChem (https://pubchem.ncbi.nlm.nih.gov/ accessed on 23 November 2023).

### 2.4. Optimization of HS-SPME Extraction by Central Composite Design (CCD) and Response Surface Methodology (RSM)

The simultaneous optimization of HS-SPME parameters, conducted with a dedicated CCD, permitted an efficient number of experiments to estimate the effect and interactions of variables [28]. DOM, commonly used in optimization of design parameters [29], was employed to obtain a good compromise among predicted response models obtained from CCD.

For the development of the CCD at three levels (−1, 0 and +1), polar DVB/CAR/PDMS and medium-polar Carboxen/PDMS fibers were selected, since, in the literature, these two coatings are more suitable for the analysis of surface-ripened cheeses [30,31,32]. The two-factor CCD consisted of 11 runs with three replicates of the central point. In Table 1, independent experimental factors (temperature and time of extraction) and design layout of runs were reported. More specifically, the values of extraction temperature were 30, 40 and 50 °C, while those of extraction time were 20, 40 and 60 min. These levels were chosen on the basis of information from the literature and our earlier knowledge [24] beyond our experience of surface-ripened cheeses analysis. As responses of the CCD, the total area (Y_1_) and the total number of VOCs (Y_2_), obtained in total ion chromatogram (TIC), were chosen [24,33].

HS-SPME mechanism is based on the equilibrium of analytes among three phases, that is, fiber coating, headspace and sample, and then the vapor pressures of VOCs greatly influence headspace microextraction [34,35]. Extraction temperature and extraction time are reported to be among the most influential factors on vapor pressures and equilibria of volatiles in the headspace [36,37]. However, although an increase in the extraction temperature can lead to an increase in the extraction rate, it can decrease the distribution constant and conversely cause a decrease in the sensitivity of the extraction process. Hence, a well-balanced compromise between sensitivity and extraction rate, with regard to the extraction temperature, could be reached [38].

Regarding DOM, in the first step, an individual desirability function (d_i_) for the two responses, Y_1_ and Y_2_, was created using the fitted models and establishing the optimization criteria. Desirability always takes values between 0 and 1, where d_i_ = 0 for an undesirable response, and d_i_ = 1 represents a completely desirable value, i.e., an ideal response. Intermediate values of d_i_ indicate more or less desirable responses [39]. A global desirability function (D) was then calculated as the geometric mean of the two d_i_ [40]. The optimal HS-SPME conditions are those corresponding to the maximum value of function D. The experimental design was studied with free software R version 4.1.1 [41].

### 2.5. Statistical Analysis

A total of 90 HS-SPME/GC–MS analyses for Brie samples and 90 analyses for Camembert cheese were carried out using the optimized HS-SPME conditions that resulted from CCD. For both types of cheese, 15 analyses were performed for each dose level and 45 for non-irradiated. A chemometric study was employed to detect the latent information from the massive data, to build discriminant analysis models and to identify the tentative markers. Specifically, an unsupervised PCA and supervised PLS-DA were applied to discriminate among X-ray-irradiated and non-irradiated Brie and Camembert cheeses. Among chemometric techniques, PCA and PLS-DA were assessed to be suitable for discrimination of X-ray irradiation treatment and dose levels too, considering different “omics” approaches [24,42,43]. PCA is an orthogonal method, which is capable of converting a set of observed correlated variables into a set of uncorrelated linear variables of the principal components [44]. PCA was performed to determine the presence of pattern recognition, trends, outliers and clustering that covered the entire sample space. PLS-DA method was performed to classify and to identify the most discriminant volatiles. The assessment of the importance of VOCs and selection of the significant variable were performed using the Variable Importance in Projection (VIP). This unsupervised approach was used to obtain a/some descriptive model/s, which allows the pattern description resulting from the relationship between the volatile compounds’ behavior and the dose levels of irradiation treatment. R software was applied to scale and analyze the data, and for all multivariate analyses [41,45].

## 3. Results and Discussions

### 3.1. HS-SPME Optimization by Central Composite Design (CCD) in Combination with a Desirability Optimization Methodology (DOM)

**Figure 1:**
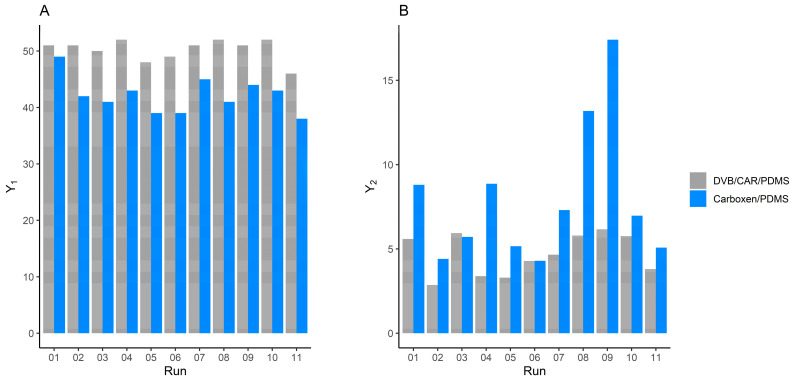
Bar plots (**A**,**B**) show the total area (Y_1_) and the total number of VOCs (Y_2_), respectively, both obtained in TIC. Y_1_ and Y_2_ were considered as responses for CCD, carried out with the two fibers DVB/CAR/PDMS (grey) and Carboxen/PDMS (light blue).

Figure 1 shows the comparison of the extraction efficiency of the DVB/CAR/PDM and Carboxen/PDMS fibers for the irradiated Brie samples in terms of the Y_1_ and Y_2_, reported, respectively, in an A and B plot. Both fibers showed similar results in terms of the Y_1_ of the extracted VOCs, while the Carboxen/PDMS showed the highest Y_2_ in all the different experimental conditions, (Figure 1B) and thus, it was chosen as the fiber for the following analyses. Successively, the optimal values of the extraction time and temperature, using the Carboxen/PDMS fiber, were selected by the DOM. The maximum D (D = 0.806) showed that the combined optimized outputs in terms of the overall response of the volatile components were obtained at 60 min and 50 °C.

### 3.2. VOC Identification and Characterization

In total, sixty-three and seventy-nine VOCs were identified for the Brie and Camembert cheeses, respectively. All the compounds were categorized into 12 classes and reported in Table S1 (Brie) and Table S2 (Camembert). The area of each compound was extracted at a selected *m*/*z* value, then normalized with respect to the area of the selected *m*/*z* of IS (*m*/*z* 59). Because HS-SPME is a partition-coefficient-based extraction technique, its quantitative application has several issues [46], so the comparison of semi-quantitative profiles based on normalized areas has been considered as the most suitable approach.

In the Brie samples, a total of nine alcohols, one aldehyde, six alkanes, four alkenes, one aromatic compound, ten carboxylic acids, two esters, ten ketones, sixteen methyl esters, two other compounds, one sulfur-containing compound and one terpene were determined, while twelve alcohols, four aldehydes, seven alkanes, four alkenes, one alkyne, two aromatic compounds, eleven carboxylic acids, nine ketones, sixteen methyl esters, two lactones, four other compounds and seven sulfur-containing compounds were found in the Camembert samples. Methyl esters and carboxylic acids, followed by alcohols and ketones, were the most abundant compounds in the Brie and Camembert, in both the non-treated and treated samples. Indeed, these classes are the main characteristic volatiles in the majority of the surface-ripened cheeses [47].

VOCs, which contribute to the typical aroma, are produced principally by lipolytic and proteolytic pathways, and by the metabolism of lactose, lactate and citrate [3]. As reported by Chen et al. [48], the process of lipolysis is ascribed to the following three pathways: (1) the residual lipoprotein lipase in bovine milk; (2) esterase activities in lactic acid bacteria; (3) the activity of lipases synthesized by molds or yeast. As lipolysis products, carboxylic acids directly impact the cheese flavor and also serve as the initial precursors of secondary catabolism reactions, resulting in the generation of various volatiles, such as esters, lactones, thioesters, secondary alcohols and methyl ketones [49].

**Figure 2:**
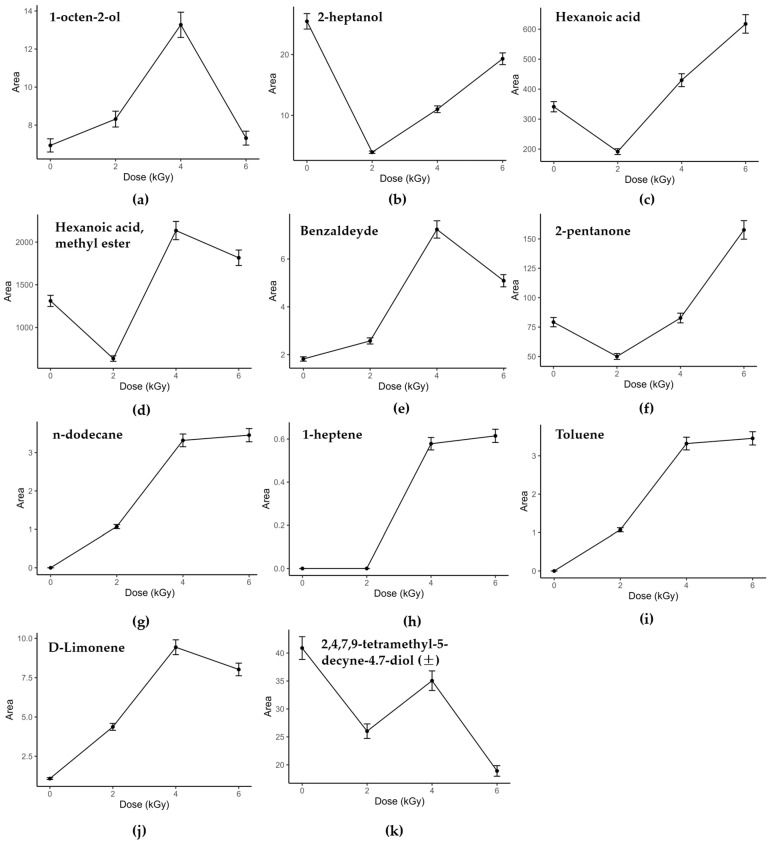
Normalized areas (mean ± standard error) of VOCs, representative for each class in Brie samples at 0 kGy (non-irradiated), 2.0 kGy, 4.0 kGy and 6.0 kGy: (**a**) 1-octen-2-ol; (**b**) 2-heptanol; (**c**) Hexanoic acid; (**d**) Hexanoic acid, methyl ester; (**e**) Benzaldeyde; (**f**) 2-pentanone; (**g**) n-dodecane; (**h**) 1-heptene; (**i**) Toluene; (**j**) D-Limonene; (**k**) 2,4,7,9-tetramethyl-5-decyne-4.7-diol (±).

**Figure 3:**
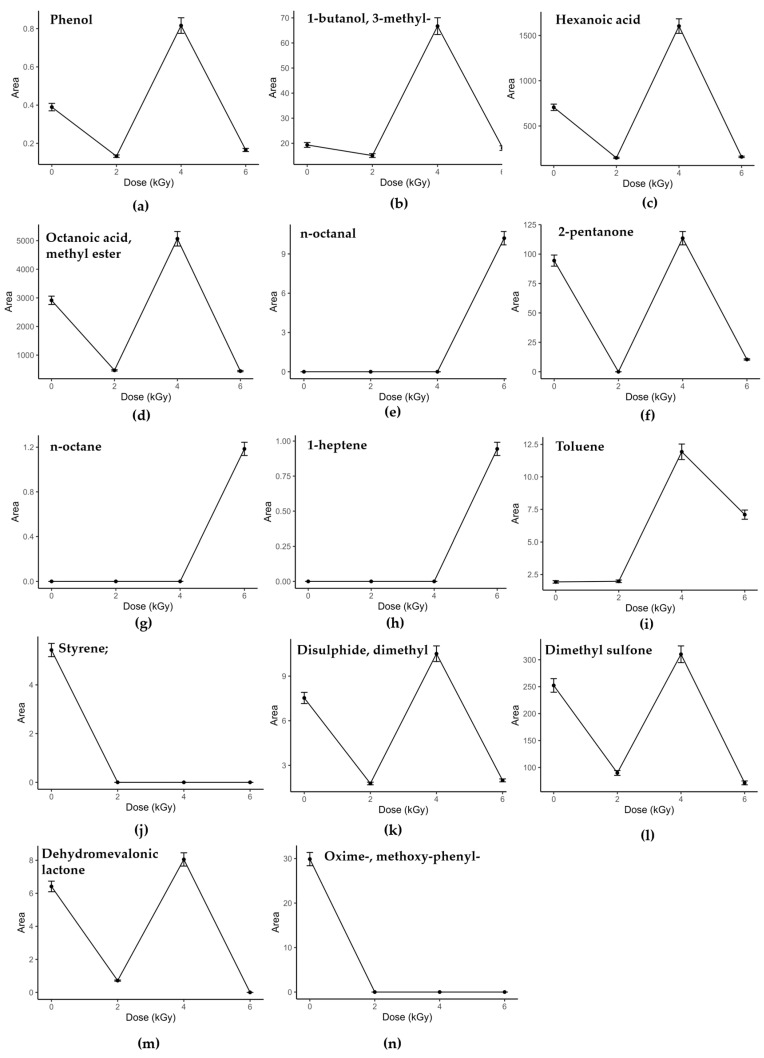
Normalized areas (mean ± standard error) of VOCs, representative for each class in Camembert samples at 0 kGy (non-irradiated), 2.0 kGy, 4.0 kGy and 6.0 kGy: (**a**) Phenol; (**b**) 1-butanol, 3-methyl-; (**c**) Hexanoic acid; (**d**) Octanoic acid, methyl ester; (**e**) n-octanal; (**f**) 2-pentanone; (**g**) n-octane; (**h**) 1-heptene; (**i**) Toluene, (**j**) Styrene; (**k**) Disulphide, dimethyl (**l**) Dimethyl sulfone; (**m**) Dehydromevalonic lactone; (**n**) Oxime-, methoxy-phenyl-.

Regarding the alcoholic class, in the Brie samples were identified nine compounds (ethanol; 1-hexanol; 1-heptanol; 1-hexanol, 2-ethyl; 2-heptanol; 2-nonanol; 1-octen-3-ol; phenol; 7-octen-2-ol), while in the Camembert, twelve (ethanol; 1-hexanol; 1-heptanol; 1-octanol; 1-butanol, 3-methyl; 1-hexanol, 2-ethyl; 2-heptanol; 2-nonanol; 2-butanol, 3-methyl; phenylethyl alcohol; phenol; 7-octen-2-ol). The primary and aromatic alcohols increased with treatment up to 2.0 kGy or 4.0 kGy, then at higher doses decreased slightly in both matrices (Figure 2a and Figure 3a,b). These compounds are the by-products of the primary oxidation of unsaturated fatty acids, and their decreased amount with higher doses is probably due to the further oxidation to aldehydes, as reported by Bliznyuk et al. [18]. The Brie secondary alcohols showed a linear behavior in the irradiated samples (Figure 2b), while in the Camembert, the trend was in line with this of the primary alcohols. These volatiles are produced by mold lipases but also by β-oxidation induced by irradiation, which favors not only their production but also their oxidation to ketones [18,50,51]. Unsaturated alcohols, namely 1-octen-3-ol and 7-octen-2-ol, responsible for the characteristic mushroom flavor [52] and present in the treated and non-treated cheeses, showed the same behavior as the aromatic and primary alcohols with respect to the treatment dose (Figure 2a). Since short-chain unsaturated alcohols arise from the catabolic metabolism of an unsaturated fat, such as linolenic acid [53], irradiation could further induce transformation with increased production compared to non-irradiated, but higher doses may degrade them.

In Brie, seven straight-chain saturated carboxylic acids (acetic acid, butanoic acid, penta-noic acid, hexanoic acid, octanoic acid, nonanoic acid, decanoic acid), two methyl-branched acids (propanoic acid, 2-methyl- and butanoic acid, 3-methyl-) and one ethyl-branched acid (hexanoic acid, 2-ethyl-) were found. In the Camembert, nine straight-chain saturated carboxylic acids (acetic acid, propanoic acid, butanoic acid, pentanoic acid, hexanoic acid, heptanoic acid, octanoic acid, nonanoic acid, decanoic acid) and two methyl-branched acids (propanoic acid, 2-methyl- and butanoic acid, 3-methyl-) were detected. Linear-chain carboxylic acids are produced from the lipolysis of triacylglycerides, diacylglycerides and monoacylglycerides [49,51]. Furthermore, carboxylic acids, formed during lipolysis, directly affect the flavor of the cheeses, but indirectly, because they are also metabolized into other highly flavored compounds, including methyl ketones, aldehydes, lactones, methyl esters and esters [47,49,51,53,54]. In the Camembert cheese, the carboxylic acids did not have a linear trend with the dose levels; indeed, they increased from 2.0 kGy up to 4.0 kGy, and then decreased slightly to 6.0 kGy (Figure 3c). Furthermore, the behavior of this class at 4.0 kGy was similar to the non-treated samples. In the Brie, on the contrary, they were augmented with increasing irradiation dose up to 6.0 kGy (Figure 2c). The branched-chain methyl and ethyl acids, which arise from the β-oxidation of fatty acids, were affected by the dose levels, as were the carboxylic acids for each cheese. For both cheeses, the qualitative composition of the fatty acids was not influenced by irradiation; meanwhile, their areas were dose-dependent. So, irradiation promotes the formation of carboxylic acids inducing lipid oxidation and lipase activity, in agreement with what has been obtained by other authors, also for different matrices [55,56,57]. However, the dose-dependent trend in the case of the Camembert cheese would lead one to the conclusion that irradiation favors the production of carboxylic acids, but at higher doses, the degradation processes exceed those of formation, as already described for alcohols.

In the Brie cheese, for the class of methyl-esters, thirteen methyl esters of straight-chain saturated carboxylic acids (acetic, butanoic, pentanoic, hexanoic, heptanoic, octanoic, nonanoic, decanoic, undecanoic, dodecanoic, tetradecanoic, pentadecanoic, hexadecanoic), one of unsaturated acid (myristoleic acid) and two of methyl-branched carboxylic acids (tridecanoic, 12-methyl- and tetradecanoic, 12-methyl-) were found. The same methyl esters of straight-chain saturated carboxylic acids found in the Brie were determined in the Camembert, plus three methyl esters of unsaturated acids (4-decenoic, cis-9-tetradecenoic and 9-hexadecenoic). The methyl esters showed the same behavior as the carboxylic acids in the two cheeses, namely, a non-linear trend as the doses increased in the Camembert (Figure 3d), while in the Brie, a slight reduction in the trend was observed among the highest doses (Figure 2d). The two esters, i.e., hexyl ester and heptyl ester of acid acetic, were found only in the Brie samples and prevailed at 4.0 kGy. The esters and methyl esters in the cheeses are produced by the esterification reaction between short-chain fatty acids and medium-long-chain fatty acids with primary and secondary alcohols produced by lactose fermentation or amino acid metabolism during fermentation [49,58,59]. The non-linear variation in these compounds, observed in relation to the irradiation dose, may be because these VOCs are by-products and intermediates of side and complex mechanisms, including those induced by radiation treatment.

Considering the aldehyde class, two benzene derivatives and two saturated short-chain aldehydes were determined, that is, benzaldehyde, phenylacetaldehyde, n-nonanal and n-decanal. Benzaldehyde was present in all the Brie and Camembert samples, including the control ones, and it increased with the dose levels, prevailing at 4.0 kGy (Figure 2e). Phenylacetaldehyde, nonanal and decanal were determined only in the Camembert at 6.0 kGy (Figure 3e). Regarding aromatic aldehydes, these compounds are mainly formed starting from α-keto acids deriving from the benzaldehyde released by the spontaneous oxidation of tryptophan and phenylalanine. This is a redox reaction, strongly influenced by temperature, since irradiation probably has the same effect, favoring catabolism acceleration [16,53]. Decanal and nonanal could be direct and indirect by-products of oxidation phenomena, i.e., the radiolysis of proteins and/or the further oxidation of primary alcohols, respectively [24,60].

The ketones class was also quite abundant in the Brie samples, where seven alkan-2-one compounds (2-propanone, 2-pentanone, 2-hexanone, 2-octanone, 2-nonanone, 2-decanone, 2-undecanone), one unsaturated alkan-2-one (8-nonen-2-one), and two other molecules with a carbonyl group in position 3 (3-octanone) and 4 (4-heptanone) were identified. In the Camembert aroma, the ketones found were seven alkan-2-one compounds (2-pentanone, 2-octanone, 2-nonanone, 2-decanone, 2-tridecanone), one unsaturated alkan-2-one (8-nonen-2-one), one alkan-3-one (3-octanone) and one aromatic ketone (acetophenone). Specifically, methyl ketones and alkan-2-ones are formed by the enzymatic β-oxidation of carboxylic acids and β-ketoacids [49,51]. In the Camembert, the ketones were abundant at 4.0 kGy and in the non-irradiated samples, while a decrease was observed at 2.0 and 6.0 kGy. This trend is probably due to the competitive reactions of ketone formation and degradation, which prevailed differently at each dose level (Figure 3f). In the Brie, this tendency, for some ketones, was less evident; indeed, at 6.0 kGy, there was not a reduction (Figure 2f).

Hydrocarbons are classified as the main radiolytic by-products of lipid oxidation, induced by irradiation treatment [16,61]. In the Brie, three straight-chain alkanes with an odd number of carbons atoms (n-heptane, n-nonane and n-undecane), three with an even number (n-hexane, n-octane and n-dodecane) and four straight-chain alkenes (1-heptene, 1-octene, 1-decene and 1-dodecene) were identified. In the Camembert, regarding the saturated short- and medium-chain alkanes, five compounds had an odd number of carbons (n-heptane, n-nonane, n-undecane, n-tridecane and n-pentadecane) and two had an even number (n-octane and n-decane). Furthermore, four alkenes (1-octene, 1-decene, 1-undecene and 1-dodecene) and one alkyne (acetonitrile) were detected. All these hydrocarbons were determined only in the irradiated samples, showing a linear trend with dose irradiation, confirming that their production was induced by treatment with X-rays (Figure 2g,h and Figure 3g,h). Among the alkanes, n-hexane was an exception, because it was also determined in the non-irradiated Brie samples. Styrene and toluene, as aromatic compounds, were also detected in the cheese samples. The former, which has a strong plastic smell, was also found only in the non-irradiated Camembert samples, while the latter was determined in all the samples (Figure 2i and Figure 3i,j). Several studies have demonstrated the presence of linear saturated alkanes and also styrene in control samples, probably derived from processing contamination or from packaging materials [61,62]. Toluene was present already in the non-irradiated samples, as an oxidation product of β-carotene [63]. On the other hand, this benzene-derived compound showed a strong dependence on dose levels; its increased production could be induced by carotene and protein degradation, correlated to irradiation [16]. The trend of toluene was similar to other classes, already discussed for the Brie and Camembert cheeses (Figure 2i and Figure 3i).

Sulfur volatiles were detected in the Camembert samples (methyl thioloacetate; 2,4-dithiapentate; hexanethioic acid, S-propyl ester; disulfide, dimethyl; dimethyl sulfone; methanethiol; ethanethiol). These sulfur-containing molecules, produced through the reaction of free fatty acids with sulfhydryl groups, impart ‘sulfurous and garlic-like’ and ‘mushroom-like’ odor notes, characteristic of Camembert-type cheeses [61,64,65]. As already discussed and observed for alcohols, carboxylic acid, methyl ester and ketone classes increased as the dose increased, due to secondary chemical reactions induced by irradiation (Figure 3k,l) [16,66]. On the other hand, the competitive mechanisms of production and degradation led to the observation of a similar behavior for the control and irradiated samples at 4.0 kGy (Figure 3k,l).

D-Limonene, characteristic of a lemon and orange aroma, was found in the Brie samples, which was affected and increased by the treatment (Figure 2j). Terpenes are widely acknowledged for their use as biomarkers that certify the origin and quality of animal products, and also for their various properties, such as antimicrobial, antifungal, antiparasitic, antiviral, anti-allergenic and anti-inflammatory [67,68]. Regarding lactones, which are fat-derived aroma compounds [53], dehydromevalonic lactone and δ-decalactone were found in the Camembert cheese. The lactones seemed to undergo the same changes due to the treatment, with competitive processes of formation and degradation (Figure 3m).

The class of other compounds was made up of VOCs that are contamination molecules, therefore, are not directly linked to the biochemical events of the cheese production or ripening process. Cheese may contain regulated disinfectant by-products, such as trichloromethane, primarily through contact with disinfectants used to clean all contact surfaces, such as processing equipment and tanks. Furthermore, the packaging material is capable of releasing various types of compounds, such as oxygen-containing compounds and plastic derivatives, through migration phenomena [62]. The trend of these VOCs, in the non-irradiated and irradiated samples, was constant or decreased with the treatment, showing that, in these products, irradiation does not induce the migration of molecules from the packaging, even at the 6.0 kGy level (Figure 2k and Figure 3j,n).

These findings have also been reported for other matrices, demonstrating how the irradiation treatment can induce the production of some volatiles, as well as, sometimes, in parallel with secondary chemical reactions, influenced by dose levels, also lead to the degradation of themselves [5,16,18,66]. Indeed, the radio-induced chemical transformations of biomolecules, such as proteins and lipids, which include conformational changes, cross-linking, aggregation, the oxidative breaking of covalent bonds, the further oxidation and formation of free radicals, are connected and influence each other [14,57,69,70].

### 3.3. Chemometric Analysis

#### 3.3.1. PCA of X-ray-Irradiated Cheeses

**Figure 4:**
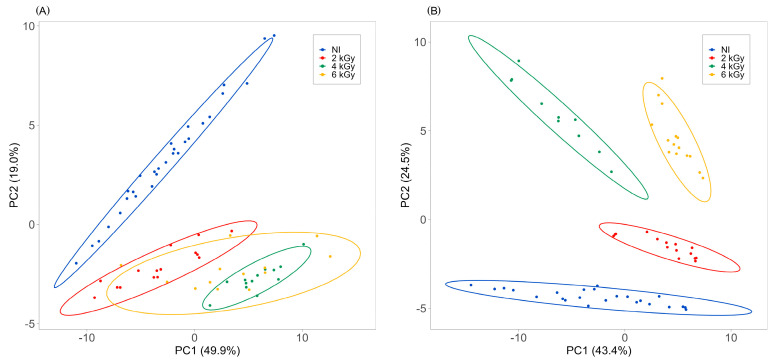
Score plots of PCA of Brie (**A**) and Camembert (**B**), blue for non-irradiated (NI), red for 2.0 kGy, green for 4.0 kGy and yellow for 6.0 kGy.

Figure 4 displays the score plot of 90 samples divided into the 2.0, 4.0 and 6.0 kGy treatments compared with the non-irradiated ones. The first two main components (PC1 and PC2) explained 49.9% and 19.0% (total value of 68.9%) and 43.4% and 24.5% (total value of 67.9%) of the variance for the Brie (Figure 4A) and Camembert (Figure 4B), respectively. In the PCA relating to the Camembert, a separation was evident between the untreated samples and treated samples, and between the different treatment doses, as shown by the four confusion ellipses (*p*-value = 0.05). For the Brie, the separation of the groups was present only between the treated and non-treated groups, while the PCA did not highlight any separation between the samples for the dose levels.

#### 3.3.2. PLS-DA Models and Permutation Test

For the building of the PLS-DA models, data pre-processing was carried out to select the VOCs as significant variables. This step was performed with the VIP score, which is a significant parameter for evaluating the contribution of a given variable to the whole model [71]. Consequently, the VOCs having a cut off value of 1.2 for the VIP score were potentially significant for the separation of the samples on the basis of irradiation. As shown in Table 2, 15 and 14 VOCs for the Camembert and Brie, respectively, were selected as important contributors, including alcohols, alkenes, aldehyde, alkynes and ketones, one sulfur compound and one terpene.

A double cross-validation algorithm [72] was used and Table 3 shows the evaluation of the performance for the two types of cheeses analyzed. In general, the PLS-DA model improves when the Q^2^, DQ^2^, accuracy, sensitivity, specificity and AUROC increase, while for the root mean squared error of cross-validation (RMSECV), which indicates how closely a model predicts the measured values, the optimal targeted value is the lowest, as reported in our previous works [24,43]. In this investigation, for a better evaluation of the model’s performance, the values of efficiency (Equation (1)), precision (Equation (2)), and the Matthews correlation coefficient (Equation (3)) were calculated. More specifically, the efficiency of a model is computed as the geometric mean of the sensitivity and specificity values; the precision is defined as the ratio between the number of samples correctly classified and the total number of samples classified by the same model:

(1) $$efficiency=\sqrt{\frac{TP\times TN}{\left(TP+FN\right)\times (TN+FP)}}$$

(2) $$precision=\frac{TP}{TP+FP}$$

where *TP* and *TN* mean “true positive” and “true negative” and correspond to the samples correctly classified. Vice versa, *FP* and *FN* mean “false positive” and “false negative” and correspond to the samples non-correctly classified. The efficiency and precision may vary between 0 and 1 [44]. Finally, the so-called Matthews correlation coefficient considers all four possible outcomes (*TP*, *TN*, *FP* and *FN*) as follows:

(3) $$Matthews\;correlation\;coefficient=\frac{TP\times TN-FP\times FN}{\sqrt{\left(TP+FP)\times (TP+FN)\times (TN+FP)\times (TN+FN\right)}}$$

The results obtained revealed the strong capacity of the PLS-DA models in discriminating the irradiated and non- irradiated samples for both cheeses, but for the Brie, as already noted with the PCA, all the calculated diagnostic parameters, particularly the low values of sensitivity, highlighted the difficulty of discriminating between the irradiated samples based on the dose. In contrast, for the Camembert, the excellent value obtained by the models for all the parameters revealed that the models also allowed discrimination between the samples treated at 2.0, 4.0 and 6.0 kGy.

**Figure 5:**
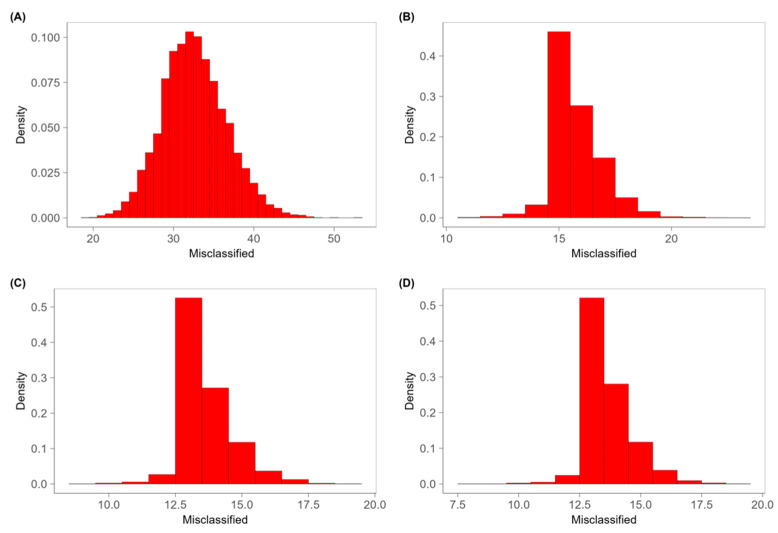
Density of misclassified samples of permutation test, for the results of (**A**) non-irradiated, (**B**) X-ray-irradiated at 2.0 kGy, (**C**) at 4.0 kGy and (**D**) at 6.0 kGy in PLS-DA model for Brie.

**Figure 6:**
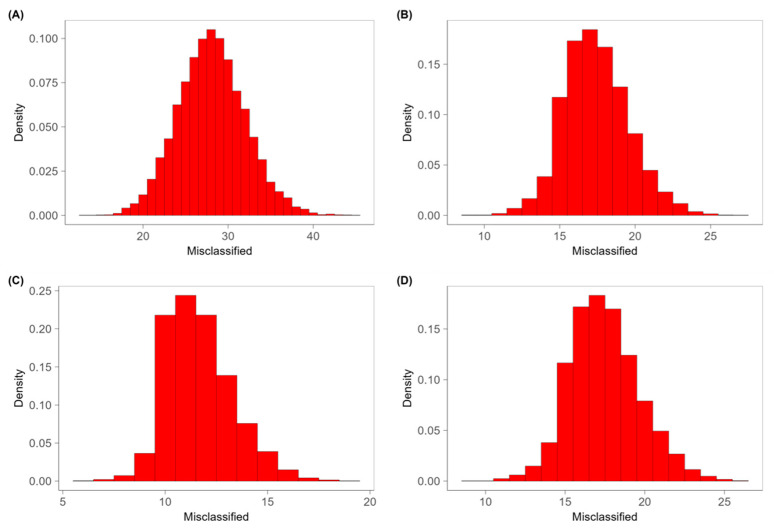
Density of misclassified samples of permutation test, for the results of (**A**) non-irradiated, (**B**) X-ray-irradiated at 2.0 kGy, (**C**) at 4.0 kGy and (**D**) at 6.0 kGy in PLS-DA model for Camembert.

These considerations are supported by the results obtained from the permutation test that allows us to verify if the results obtained in the validation of the classification models depend on the casual case. The classification of the treatments was randomly permuted and associated with the volatile profile of the samples. A PLS-DA model was built with this new dataset, and it identified the number of misclassified samples with cross-validation methods. This process was iterated 30.000 times and a distribution for the misclassified samples was obtained. If the experimental data are characteristic of a given treatment, the distribution obtained in the permutation test is expected to be binomial with *p* = 0.5 (*p* is the probability of misclassifying a sample) [43]. As suggested by Figure 5A and Figure 6A, for both cheeses, the distributions of the misclassified non-irradiated and irradiated samples in the permuted dataset were compatible with the mean value of a binomial distribution with probability π = 0.5, confirming that the discriminating ability of the models was not determined by random phenomena. Considering instead the various doses of treatment, while the Camembert continues to maintain a binomial distribution with probability π = 0.5 (Figure 6B–D), the Brie samples treated at the different doses presented an asymmetrical distribution, highlighting that the model was not adapted to discriminate on the dose (Figure 5B–D).

## 4. Conclusions

The present work gives important insight about the X-ray irradiation effects on the volatile profiles of two surface-ripened cheeses, that is, Brie and Camembert. A volatolomic strategy was applied using the HS-SPME/GC–MS procedure. By means of a dedicated CCD together with the DOM, the best conditions for the HS-SPME procedure, considering the fiber type, extraction time and temperature, were determined. The optimized parameters, resulting at 60 min and 50 °C with the Carboxen/PDMS fiber, were used to analyze the Brie and Camembert samples at different irradiation doses (2.0, 4.0 and 6.0 kGy), including the non-irradiated samples. The quality-type fingerprints of the Brie and Camembert cheeses were characterized in detail, highlighting that the aroma profile was influenced by irradiation. The aldehydes and hydrocarbons increased with the irradiation dose. Furthermore, the results showed a non-linear trend in relation to the dose levels, for the primary alcohols, methyl esters, carboxylic acids, ketones, lactones and sulfur-containing compounds, increasing up to 4.0 kGy, and then decreased slightly at 6.0 kGy. In summary, the main variations in the volatile profiles were encountered at 2.0 kGy for the Brie, and at 2.0 and 6.0 kGy for the Camembert, suggesting that these doses were the most effective in promoting degradation mechanisms. Irradiation, with its direct and indirect effects on biomolecules present in cheese matrices, in particular, lipids and proteins, can induce multiple oxidation mechanisms that interact with each other, where the production and degradation of the same volatile compounds could be competitive, leading to non-linear dose-dependent behavior. Hence, as for other food products, even for cheeses, it can be stated that the framework of the radio-induced modifications on the volatile profiles is very complex. No migration of molecules from the packaging was observed. The score plots and the models, obtained by PCA and PLS-DA, highlighted the excellent discrimination of the control with respect to the treated samples, identifying as potential markers 15 and 14 VOCs for the Camembert and Brie, respectively. On the other hand, for the Camembert, an optimal discrimination of the samples based on the treatment dose was obtained, while for the Brie, no separation for the dose levels was observed. This innovative research contributes to the understanding of how X-ray irradiation impacts the volatolomic composition of surface-ripened cheeses, providing valuable insights for both the acceptability for consumers and for developing food safety control plans. Finally, integrating the volatolomic approach with other omic methods, such as lipidomics and proteomics, could allow for insights into X-ray irradiation treatment on dairy products.

## Figures and Tables

**Table 1:** Experimental matrix of CCD for the two factors with coded and real value assignments.

Factors	Factor Levels			
		−α	0	α
Extraction temperature (°C)		30	40	50
Extraction time (minutes)		20	40	60
RUN	X_1_	X_2_	Extraction temperature (°C)	Extraction time (minutes)
1	0	1	40	60
2	−1	1	30	60
**3**	**0**	**0**	**40**	**40**
**4**	**0**	**0**	**40**	**40**
5	−1	0	30	40
6	0	−1	40	20
**7**	**0**	**0**	**40**	**40**
8	1	0	50	40
9	1	1	50	60
10	1	−1	50	20
11	−1	−1	30	20

The three replicates of the central points are reported in bold.

**Table 2:** List of potential VOCs for discrimination of irradiated and non-irradiated samples used in PLS-DA models at a cut off value of 1.2.

Class	VOC	Cheese
Alcohols	1-hexanol	CAMEMBERT/BRIE
Alcohols	1-heptanol	CAMEMBERT/BRIE
Aldehydes	Benzaldehyde	CAMEMBERT
Alkanes	Toluene	CAMEMBERT
Alkanes	n-octane	CAMEMBERT/BRIE
Alkanes	n-nonane	CAMEMBERT/BRIE
Alkanes	n-undecane	CAMEMBERT
Alkanes	n-heptane	CAMEMBERT/BRIE
Alkenes	1-octene	CAMEMBERT/BRIE
Alkenes	1-dodecene	CAMEMBERT/BRIE
Alkenes	1-heptene	BRIE
Alkenes	1-undecene	CAMEMBERT
Alkenes	1-decene	BRIE
Alkynes	Acetonitrile	CAMEMBERT
Esters	Acetic acid, heptyl ester	BRIE
Ketones	3-octanone	BRIE
Ketones	2-heptanone	CAMEMBERT
Ketones	2-undecanone	CAMEMBERT/BRIE
Ketones	4-heptanone	BRIE
Sulfur compounds	2,4-dithiapentane	CAMEMBERT
Terpenes	D-Limonene	BRIE

**Table 3:** Diagnostic statistics for PLS-DA models for Brie and Camembert cheeses.

**Brie** **PLS-DA**	**Mean**				**Median**			
	**Non-Irradiated**	**2.0 kGy**	**4.0 kGy**	**6.0 kGy**	**Non-Irradiated**	**2.0 kGy**	**4.0 kGy**	**6.0 kGy**
RMSECV	0.967	1.121	0.568	0.674	0.879	1.013	0.541	0.631
Q^2^	−0.057	−1.054	0.450	0.205	0.205	−0.523	0.516	0.342
DQ^2^	0.639	0.538	0.630	0.611	0.645	0.550	0.639	0.620
Accuracy	0.889	0.888	0.931	0.926	0.886	0.886	0.929	0.929
Sensitivity	0.990	0.503	0.829	0.632	1.000	0.467	0.846	0.615
Specificity	0.818	0.993	0.955	0.993	0.829	1.000	0.947	1.000
Efficiency	0.900	0.704	0.888	0.790	0.897	0.683	0.895	0.784
Precision	0.794	0.959	0.812	0.954	0.800	1.000	0.800	1.000
Matthews	0.797	0.643	0.777	0.738	0.794	0.638	0.771	0.752
AUROC	0.980	0.975	0.975	0.970	0.982	0.978	0.977	0.973
**Camembert** **PLS-DA**	**Mean**				**Median**			
	**Non-irradiated**	**2.0 kGy**	**4.0 kGy**	**6.0 kGy**	**Non-irradiated**	**2.0 kGy**	**4.0 kGy**	**6.0 kGy**
RMSECV	0.590	0.659	0.278	0.215	0.586	0.650	0.272	0.210
Q2	0.628	0.391	0.853	0.935	0.634	0.412	0.859	0.939
DQ2	0.717	0.543	0.894	0.973	0.721	0.546	0.895	0.973
Accuracy	0.976	0.975	0.995	1.000	0.984	0.984	1.000	1.000
Sensitivity	0.948	0.951	0.966	1.000	0.958	1.000	1.000	1.000
Specificity	0.994	0.982	1.000	1.000	1.000	0.980	1.000	1.000
Efficiency	0.970	0.965	0.982	1.000	0.979	0.990	1.000	1.000
Precision	0.990	0.942	1.000	1.000	1.000	0.938	1.000	1.000
Matthews	0.950	0.929	0.980	1.000	0.967	0.958	1.000	1.000
AUROC	0.998	0.995	1.000	1.000	0.999	0.998	1.000	1.000

## Data Availability

Data is contained within the article or Supplementary Materials.

## Supplementary Materials

The following supporting information can be downloaded at https://www.mdpi.com/article/10.3390/foods13030416/s1, Table S1: List of VOCs identified in non-treated and X-ray-irradiated Brie samples, at three dose levels of 2.0, 4.0 and 6.0 kGy. Table S2: List of VOCs identified in non-treated and X-ray-irradiated Camembert samples, at three dose levels of 2.0, 4.0 and 6.0 kGy.
