# Supplementary material for: Headspace Solid-Phase Microextraction/Gas Chromatography–Mass Spectrometry and Chemometric Approach for the Study of Volatile Profile in X-ray Irradiated Surface-Ripened Cheeses
Source: Foods. 2024 Jan 27;13(3):416. doi: 10.3390/foods13030416 (PMC10855764; doi:10.3390/foods13030416)
Supplement: Supplementary file 1 [file foods-13-00416-s001.zip › foods-2824027-supplementary.pdf]

Supplementary material of article:

Headspace Solid-Phase Microextraction/Gas Chromatography–Mass Spectrometry and Chemometric Approach for the Study of Volatile Profile in X-Ray Irradiated Surface-Ripened Cheeses

Table S1: List of VOCs identified in non-treated and X-ray-irradiated Brie samples, at three dose levels of 2.0, 4.0 and 6.0 kGy.

**Class**	**VOCs**	**CAS**	**MW**	**m/z**	**RT**	**LRI$_E$**	**LRI$_R$**	**Odor description**
Alcohols	Ethanol	64-17-5	46	45	8.9	905	905	Vinous, alcoholic
	2-heptanol	543-49-7	116	45	23.3	1305	1304.4	Cytrus, herbal, lemon grass
	1-hexanol	111-27-3	102	56	24.5	1311	1311	Ethereal fuel oil fruity alcoholic sweet green, fruity, apple-skin and oily
	1-octen-3-ol	3391-86-4	128	57	27.6	1410	1410	Mushroom
	1-heptanol	111-70-6	116	70	27.9	1411	1419	Musty leafy violet herbal green sweet woody peony
	1-hexanol, 2-ethyl	104-76-7	130	57	28.9	1417	1441	Solvent-like, fermented with oily nutty and fatty notes and a slight green aldehydic under note
	2-nonanol	628-99-9	144	45	29.9	1504.0	1505	Citrus fresh floral oily sweet
	7-octen-2-ol	39546-75-3	128	45	31.5	1514	nd	Waxy green orange aldehydic rose mushroom
	Phenol	108-95-2	94	66	44.0	2004	2004	Phenolic plastic rubber
Aldehyde	Benzaldehyde	100-52-7	106	106	30.6	1508	1508	Strong sharp sweet bitter almond cherry
Alkanes	n-hexane	110-54-3	86	57	4.3	-*	600	Petroleum-like
	n-heptane	142-82-5	100	43	4.7	701	700	Petroleum-like
	n-octane	111-65-9	114	43	5.7	707	731	Petroleum-like
	n-nonane	111-84-2	128	57	7.8	900	900	Petroleum-like
	n-undecane	1120-21-4	156	57	14.4	1019.2	1100	Petroleum-like
	n-dodecane	112-40-3	170	57	17.4	1112.9	1200	Petroleum-like
Alkenes	1-heptene	592-76-7	98	56	5.1	729	736	Petroleum-like
	1-octene	111-66-0	112	55	6.5	804	822	Petroleum-like
	1-decene	872-05-9	140	41	12.6	1011	1020	Mild odor of petroleum
	1-dodecene	112-41-4	168	55	18.8	1201	1231.6	Mild odor of petroleum-like
Aromatic compounds	Toluene	108-88-3	92	91	12.9	1013	1012	Sweet
Carboxylic acids	Acetic acid	64-19-7	60	43	28.2	1413	1413	Sharp pungent sour vinegar
	Propanoic acid, 2-methyl	79-31-2	88	43	31.8	1515	1520	Acidic sour cheese dairy buttery rancid

	n-butanoic acid	107-92-6	88	60	33.7	1609	1609	Sharp acetic cheese butter fruit
	Butanoic acid, 3-methyl-	503-74-2	102	60	34.8	1615	1621	Sour stinky feet sweaty cheese tropical
	n-pentanoic acid	109-52-4	102	60	36.8	1710	1711	Sickening putrid acidic sweaty rancid
	n-hexanoic acid	142-62-1	130	60	39.8	1811	1812	Sour fatty sweat cheese
	Hexanoic acid, 2-ethyl-	149-57-5	144	88	42.3	1910	1910	Mild odor
	n-octanoic acid	124-07-2	144	60	45.2	2011	2011	Fatty waxy rancid oily vegetable cheesy
	n-nonanoic acid	112-05-0	158	60	47.7	2111	2119	Waxy dirty cheese cultured dairy
	n-decanoic acid	334-48-5	172	60	50.2	2212	2227	Rancid sour fatty citrus
Esters	Acetic acid, hexyl ester	142-92-7	144	43	21.1	1215	1240	Green, fruity, sweet, fatty, fresh, apple and pear
	Acetic acid, heptyl ester	112-06-1	158	43	24.8	1313	1360	Green, waxy, fatty, citrus, aldehydic, winey and woody
Ketones	2-propanone	67-64-1	58	43	6.1	802	802	Solvent ethereal, apple, pear
	2-pentanone	107-87-9	86	43	10.5	1002	1001	Fingernail polish
	4-heptanone	123-19-3	114	71	15.8	1135	1135	Fruity, cheesy, sweet, cognac, pineapple
	2-hexanone	591-78-6	100	43	14.2	1019	1060	Fruity, fungal, meaty, buttery
	3-octanone	106-68-3	128	57	20.6	1210	1210	Herbal, musty, mushroom
	2-octanone	111-13-7	128	43	21.9	1217	1262	Earthy, musty
	2-nonanone	821-55-6	142	58	25.5	1317	1342	Fresh, sweet green weedy earthy herbal
	8-nonen-2-one	5009-32-5	140	43	27.6	1410	1473	Fruity baked
	2-decanone	693-54-9	156	58	29.1	1418	1463	Orange floral fatty peach
	2-undecanone	112-12-9	170	58	32.3	1518	1564	Waxy fruity creamy fatty orris floral
Methyl esters	Acetic acid, methyl ester	79-20-9	74	43	6.3	803	804	Sour
	Butanoic acid, methyl ester	623-42-7	102	43	10.7	1003	1001	Fruit, apple,sweet, banana, pineapple
	Pentanoic acid, methyl ester	624-24-8	116	74	14.4	1020	1048	Sweat
	Hexanoic acid, methyl ester	106-70-7	130	74	18.0	1116	1142	Fruity, apricot, strawberry, pineapple
	Heptanoic acid, methyl ester	106-73-0	144	74	21.8	1216	1241	Fruity, sweet, waxy apple
	Octanoic acid, methyl ester	111-11-5	158	74	25.3	1316	1352	Fatty waxy rancid oily vegetable cheesy
	Nonanoic acid, methyl ester	1731-84-6	172	74	29.0	1418	1476	Sweet fruity pear waxy tropical wine
	Decanoic acid, methyl ester	110-42-9	186	74	32.0	1517	1550	Oily wine fruity floral
	Undecanoic acid, methyl ester	1731-86-8	200	74	35.4	1700	1699	Fatty waxy fruity
	Dodecanoic acid, methyl ester	111-82-0	214	74	38.4	1802	1801	Waxy soapy creamy coconut mushroom
	Tridecanoic, 12-methyl-, methyl ester	5129-58-8	242	74	42.6	1912	-**	-***
	Tetradecanoic acid, methyl ester	24-10-7	242	74	43.9	2002	2001	Fatty waxy petal
	Myristoleic acid, methyl ester	56219-06-8	240	55	44.6	2008	2000	Fatty waxy petal

	Tetradecanoic, 12-methyl-, methyl ester	5129-66-8	256	74	45.5	2013	-**	-***
	Pentadecanoic acid, methyl ester	7132-64-1	256	74	46.3	2103	2102	Waxy, soapy, creamy, coconut, with mushroom nuances
	Hexadecanoic acid, methyl ester	112-39-0	270	74	48.8	2204	2204	Oily waxy fatty orris
Other compounds	2,4,7,9-tetramethyl-5-decyne-4.7-diol (+)	126-86-3	226	109	45.6	2014	-**	Odorless
	2,4,7,9-tetramethyl-5-decyne-4.7-diol (-)	126-86-3	226	109	45.8	2015	-**	Odorless
Sulfur-contain compounds	Dimethyl sulfone	67-71-0	94	79	41.5	1905	1906	Sulfurous burnt
Terpene	D-Limonene	5989-27-5	136	68	17.9	1115	-**	Citric, lively, nuances of tangerine, celery and lemon oil

MW: molecular weight; m/z: fragment ion with the higher relative intensity; RT: retention time; LRI_E: experimental values of linear retention index; LRI_R: reference values of linear retention index; -* not included in the RTs range of the C7-C30 standard solution; -**not available on PubChem (https://pubchem.ncbi.nlm.nih.gov) -*** not available on PubChem (https://pubchem.ncbi.nlm.nih.gov) and on The Good Scents Company Information System (http://www.thegoodscentscompany.com/search2.html).

Table S2: List of VOCs identified in non-treated and X-ray-irradiated Camembert samples, at three dose levels of 2.0, 4.0 and 6.0 kGy.

**Class**	**VOCs**	**CAS**	**PM**	**m/z**	**RT**	**LRI_E**	**LRI_R**	**Odor**
Alcohols	Ethanol	64-17-5	46	45	8.9	905	905	Vinous, alcoholic
	2-butanol, 3-methyl	598-75-4	88	45	16.1	1106	1108	Musty, alcoholic, with nuances of vegetables
	1-butanol, 3-methyl-	123-51-3	88	55	19.6	1205	1204	Alcoholic, fruity,cognac, molasses
	2-heptanol	543-49-7	116	45	23.3	1305	1304.4	Cytrus, herbal, lemon grass
	1-hexanol	111-27-3	102	56	24.5	1311	1311	Ethereal fuel oil fruity alcoholic sweet green, fruity, apple-skin and oily
	1-heptanol	111-70-6	116	70	27.9	1411	1419	Musty leafy violet herbal green sweet woody peony
	1-hexanol, 2-ethyl	104-76-7	130	57	28.9	1417	1441	Solvent-like, fermented with oily nutty and fatty notes and a slight green aldehydic under note
	2-nonanol	628-99-9	144	45	29.9	1504.0	1505	Citrus fresh floral oily sweet
	1-octanol	111-87-5	130	56	31.0	1511	1510	Waxy green creamy citrus orange cheese fruity
	7-octen-2-ol	39546-75-3	128	45	31.5	1514	nd	Waxy green orange aldehydic rose mushroom
	Phenylethyl Alcohol	60-12-8	122	91	41.6	1905	1905	Floral rose dried rose flower rose water
	Phenol	108-95-2	94	66	44.0	2004	2004	Phenolic plastic rubber
Aldehydes	n-octanal	124-13-0	128	43	21.6	1215	1247	Aldehydic, fat, soap, lemon

	n-decanal	112-31-2	156	43	28.9	1417	1448	Sweet aldehydic waxy orange peel citrus floral
	Benzaldehyde	100-52-7	106	106	30.6	1508	1508	Strong sharp sweet bitter almond cherry
	Phenylacetaldehyde	122-78-1	120	91	34.9	1616	1616	Green sweet floral hyacinth clover honey cocoa
Alkanes	n-heptane	142-82-5	100	43	4.7	701	700	Petroleum-like
	n-octane	111-65-9	114	43	5.7	707	731	Petroleum-like
	n-nonane	111-84-2	128	57	7.8	900	900	Petroleum-like
	n-undecane	1120-21-4	156	57	14.4	1019.2	1100	Petroleum-like
	n-dodecane	112-40-3	170	57	17.4	1112.9	1200	Petroleum-like
	n-tridecane	629-50-5	184	57	21.4	1213.9	1300	Petroleum-like
	n-pentadecane	629-62-9	212	57	28.8	1416.5	1500	Petroleum-like
Alkenes	1-octene	111-66-0	112	55	6.5	804	822	Petroleum-like
	1-decene	872-05-9	140	41	12.6	1011	1020	Mild odor of petroleum
	1-undecene	821-95-4	154	55	15.8	1105	1135	Mild odor of petroleum-like
	1-dodecene	112-41-4	168	55	18.8	1201	1231.6	Mild odor of petroleum-like
Alkynes	Acetonitrile	75-05-8	41	41	11.6	1007	1006.4	Aromatic, ethereal
Aromatic compounds	Toluene	108-88-3	92	91	12.9	1013	1012	Sweet
	Styrene	100-42-5	104	104	20.8	1211	1228.7	Balsamic, almond
Carboxylic acids	Acetic acid	64-19-7	60	43	28.2	1413	1413	Sharp pungent sour vinegar
	Propanoic acid	79-09-4	74	74	30.9	1510	1510	Pungent acidic cheesy vinegar
	Propanoic acid, 2-methyl	79-31-2	88	43	31.8	1515	1520	Acidic sour cheese dairy buttery rancid
	Butanoic acid	107-92-6	88	60	33.7	1609	1609	Sharp acetic cheese butter fruit
	Butanoic acid, 3-methyl-	503-74-2	102	60	34.8	1615	1621	Sour stinky feet sweaty cheese tropical
	Pentanoic acid	109-52-4	102	60	36.8	1710	1711	Sickening putrid acidic sweaty rancid
	Hexanoic acid	142-62-1	130	60	39.8	1811	1812	Sour fatty sweat cheese
	Heptanoic acid	111-14-8	130	60	42.5	1911	1913	Rancid sour cheesy sweat
	Octanoic acid	124-07-2	144	60	45.2	2011	2011	Fatty waxy rancid oily vegetable cheesy
	Nonanoic acid	112-05-0	158	60	47.7	2111	2119	Waxy dirty cheese cultured dairy
	n-Decanoic acid	334-48-5	172	60	50.2	2212	2227	Rancid sour fatty citrus
Ketones	2-pentanone	107-87-9	86	43	10.5	1002	1001	Fingernail polish
	3-octanone	106-68-3	128	57	20.6	1210	1210	Herbal, musty, mushroom
	2-octanone	111-13-7	128	43	21.9	1217	1262	Earthy, musty

	2-nonanone	821-55-6	142	58	25.5	1317	1342	Fresh, sweet green weedy earthy herbal
	8-nonen-2-one	5009-32-5	140	43	27.6	1410	1473	fruity baked
	2-decanone	693-54-9	156	58	29.1	1418	1463	Orange floral fatty peach
	2-undecanone	112-12-9	170	58	32.3	1518	1564	Waxy fruity creamy fatty orris floral
	Acetophenone	98-86-2	120	105	34.5	1613	1615	Sweet pungent hawthorn mimosa almond acacia chemical
	2-tridecanone	593-08-8	198	58	38.65	1803	1803	Fatty waxy dairy milky coconut nutty herbal earthy
Methyl esters	Acetic acid, methyl ester	79-20-9	74	43	6.3	803	804	Sour
	Butanoic acid, methyl ester	623-42-7	102	43	10.7	1003	1001	Fruit, apple,sweet, banana, pineapple
	Pentanoic acid, methyl ester	624-24-8	116	74	14.4	1020	1048	Sweat
	Hexanoic acid, methyl ester	106-70-7	130	74	18.0	1116	1142	Fruity, apricot, strawberry, pineapple
	Heptanoic acid, methyl ester	106-73-0	144	74	21.8	1216	1241	Fruity, sweet, waxy apple
	Octanoic acid, methyl ester	111-11-5	158	74	25.3	1316	1352	Fatty waxy rancid oily vegetable cheesy
	Nonanoic acid, methyl ester	1731-84-6	172	74	29.0	1418	1476	Sweet fruity pear waxy tropical wine
	Decanoic acid, methyl ester	110-42-9	186	74	32.0	1517	1550	Oily wine fruity floral
	4-decenoic acid, methyl ester	93979-14-7	184	74	34.0	1610	1611	Fruity, pear, mango, fishy, peach, skin green
	Undecanoic acid, methyl ester	1731-86-8	200	74	35.4	1700	1699	Fatty waxy fruity
	Dodecanoic acid, methyl ester	111-82-0	214	74	38.4	1802	1801	Waxy soapy creamy coconut mushroom
	Tetradecanoic acid, methyl ester	124-10-7	242	74	43.9	2002	2001	Fatty waxy petal
	Myristoleic acid, methyl ester	56219-06-8	240	55	44.6	2008	2000	Fatty waxy petal
	Pentadecanoic acid, methyl ester	7132-64-1	256	74	46.3	2103	2102	Waxy, soapy, creamy, coconut, with mushroom nuances

	Hexadecanoic acid, methyl ester	112-39-0	270	74	48.8	2204	2204	Oily waxy fatty orris
	9-Hexadecenoic acid, methyl ester	10030-74-7	268	55	49.4	2207	2236	-***
Other compounds	2,4,7,9-tetramethyl-5-decyne-4.7-diol (+)	126-86-3	226	109	45.6	2014	-**	Odorless
	2,4,7,9-tetramethyl-5-decyne-4.7-diol (-)	126-86-3	226	109	45.8	2015	-**	Odorless
	Trichloromethane	67-66-3	118	83	12.1	1009	1010	Sweet
	Oxime-, methoxy-phenyl-	67160-14-9	151	133	37.0	1711	1710	Balsamic
Lactones	Dehydromevalonic lactone	2381-87-5	112	82	44.8	2009	1978	Sweet caramel-like
	δ-Decalactone	705-86-2	170	99	48.7	2203	2203	Fresh sweet oily coconut fruity peach creamy dairy
Sulfur-contain compounds	Methanethiol	74-93-1	48	47	4.6	700	700	Sulfur, gasoline, garlic
	Ethanethiol	75-08-1	62	62	5.2	704	731	Thiamin
	Methyl thioacetate	1534-08-3	90	43	13.2	1014	1041	Sulfours eggy cheese dairy vegetable cabbage
	Disulfide, dimethyl	624-92-0	94	94	14.2	1019	1036	Garlic
	2,4-dithiapentane	1618-26-4	108	61	22.2	1218	1256	Sulfurous, metallic, spicy
	Hexanethioic acid, S-propyl ester	2432-78-2	174	99	26.6	1404	1303	Odorless
	Dimethyl sulfone	67-71-0	94	79	41.5	1905	1906	Sulfurous burnt

MW: molecular weight; m/z: fragment ion with the higher relative intensity; RT: retention time; LRI_E: experimental values of linear retention index; LRI_R: reference values of linear retention index; -* not included in the RTs range of the C7-C30 standard solution; -**not available on PubChem (https://pubchem.ncbi.nlm.nih.gov) -*** not available on PubChem (https://pubchem.ncbi.nlm.nih.gov) and on The Good Scents Company Information System (http://www.thegoodscentscompany.com/search2.html).
